# Effect of Abaloparatide vs Alendronate on Fracture Risk Reduction in Postmenopausal Women With Osteoporosis

**DOI:** 10.1210/clinem/dgz162

**Published:** 2019-11-01

**Authors:** Benjamin Z Leder, Bruce Mitlak, Ming-yi Hu, Gary Hattersley, Richard S Bockman

**Affiliations:** 1 Endocrinology Unit, Massachusetts General Hospital and Department of Medicine, Harvard Medical School, Boston, USA; 2 Radius Health, Inc, Waltham, MA, USA; 3 Joan and Sanford Weill Medical College of Cornell University, Hospital for Special Surgery, New York, NY, USA

**Keywords:** abaloparatide, alendronate, osteoporosis, vertebral fractures, nonvertebral fractures

## Abstract

**Context:**

The ACTIVE study demonstrated the antifracture efficacy of abaloparatide in postmenopausal women with osteoporosis. ACTIVExtend demonstrated sustained fracture risk reduction with alendronate in abaloparatide-treated participants from ACTIVE. A direct comparison of the efficacy of abaloparatide and antiresorptive therapies has not been performed.

**Objective:**

The objective of this analysis is to compare the antifracture efficacy of abaloparatide in ACTIVE with that of alendronate in ACTIVExtend.

**Design:**

In this post hoc analysis, the rate of new vertebral fractures for women in ACTIVExtend (N = 1139) was calculated based on baseline and endpoint radiographs for placebo or abaloparatide in ACTIVE and alendronate in ACTIVExtend. Vertebral fracture rates between abaloparatide and alendronate were compared in a Poisson regression model. Fracture rates for nonvertebral and clinical fractures were compared based on a Poisson model during 18 months of abaloparatide or placebo treatment in ACTIVE and 18 months of alendronate treatment in ACTIVExtend.

**Results:**

The vertebral fracture rate was lower during abaloparatide treatment in ACTIVE (0.47 fractures/100 patient-years) than alendronate treatment in ACTIVExtend (1.66 fractures/100 patient-years) (relative risk reduction 71%; *P* = .027). Although the comparisons did not meet statistical significance, after switching from placebo (ACTIVE) to alendronate (ACTIVExtend), the rate of new vertebral fractures decreased from 2.49 to 1.66 fractures per 100 patient-years, and after switching from abaloparatide to alendronate from 0.47 to 0.19 fractures per 100 patient-years. The rates of nonvertebral fractures and clinical fractures were not significantly different.

**Conclusion:**

Initial treatment with abaloparatide may result in greater vertebral fracture reduction compared with alendronate in postmenopausal women with osteoporosis.

Fragility fractures are the major clinical consequence of osteoporosis and are associated with increased health-care resource utilization, morbidity and mortality, loss of independence, and functional decline (1–3). Because of the substantial burden of osteoporotic fractures, both to individuals and to society, prevention of fragility fractures is a key goal in the treatment and management of patients with osteoporosis.

Osteoporosis is caused by an imbalance between bone resorption and bone formation, resulting in the loss of bone mass and reduced bone strength (4, 5). Current pharmacologic therapies to reduce the risk of fracture in patients with osteoporosis include both antiresorptive and anabolic agents (6, 7). Antiresorptive therapies, including bisphosphonates (ie, alendronate, risedronate, ibandronate, and zoledronate) and denosumab, increase bone mineral density (BMD) and reduce fracture risk by inhibiting osteoclast-mediated bone resorption (6, 7). Conversely, anabolic drugs increase BMD and reduce fracture risk primarily by activating osteoblast function to stimulate bone formation, hence increasing BMD and improving skeletal microarchitecture (8).

Abaloparatide is a 34-amino acid synthetic analogue of the human parathyroid hormone–related protein that favors the stimulation of bone formation (9). In the 18-month, phase 3 Abaloparatide Comparator Trial In Vertebral Endpoints (ACTIVE) study, abaloparatide significantly decreased the risk of vertebral and nonvertebral fractures vs placebo in postmenopausal women with osteoporosis (10). Abaloparatide-treated participants from ACTIVE were then treated with the antiresorptive alendronate for 24 months in the ACTIVExtend study and experienced continued antifracture benefit compared with participants treated with placebo followed by alendronate, demonstrating the effectiveness of sequential treatment with abaloparatide followed by alendronate (11).

There are limited data comparing the efficacy of anabolic therapy with an antiresorptive. Recently, it was shown that the risk of new vertebral and clinical fractures is significantly lower in postmenopausal women with severe osteoporosis receiving teriparatide than in those receiving risedronate (12) as well as for those receiving romosozumab compared with alendronate (13). However, the efficacy of abaloparatide compared with alendronate, or other antiresorptive agents, has not been examined. In this post hoc analysis from ACTIVE and ACTIVExtend, we indirectly evaluated the relative antifracture efficacy of abaloparatide vs alendronate in postmenopausal women with osteoporosis.

## Methods

### Study population

The 18-month, phase 3 ACTIVE study (clinicaltrials.gov identifier: NCT01343004) enrolled 2463 postmenopausal women ages 49 to 86 years with osteoporosis at 28 study centers (10). Osteoporosis was defined based on prior radiographic vertebral fracture or recent nonvertebral fracture (within the past 5 years prior to enrollment) and a BMD T-score less than or equal to −2.5 and greater than −5.0 at the lumbar spine or femoral neck in women ages 65 years or younger. Women older than 65 years were eligible if they met the fracture criteria and had a lumbar spine or femoral neck BMD T-score of less than or equal to −2.0 and greater than −5.0 or had no prior fracture, but had a lumbar spine or femoral neck BMD T-score of less than or equal to −3.0 and greater than −5.0. Key exclusion criteria included use of bisphosphonates for more than 3 months in the past 5 years or denosumab within the past year. Additional inclusion and exclusion criteria have been previously described (10).

Women randomly assigned to the abaloparatide or placebo arm in ACTIVE who completed the end-of-treatment visit were eligible for enrollment in the ACTIVExtend extension study (clinicaltrials.gov identifier: NCT01657162). Additional criteria for inclusion in the extension have been previously described (11, 14).

### Study designs

**Figure 1.**
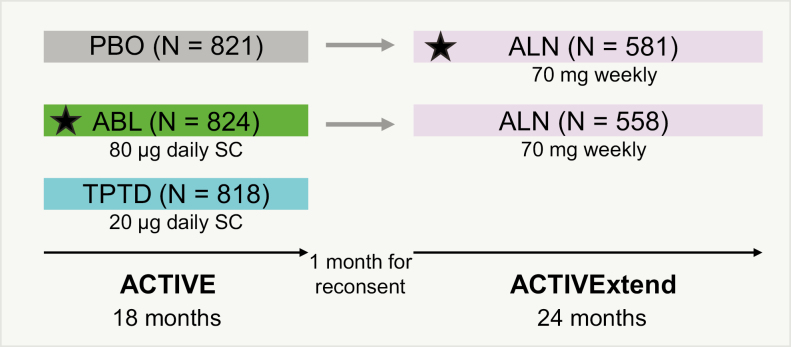
Study design for ACTIVE and ACTIVExtend. Women were randomly assigned 1:1:1 to double-blind abaloparatide (80 μg/d), matching placebo, or open-label teriparatide (20 μg/d) for 18 months. Women from the abaloparatide and placebo groups were eligible to enter the 24-month extension, during which they were treated with alendronate (70 mg/d). Stars indicate the treatment groups being compared in this analysis. ABL, abaloparatide; ACTIVE, Abaloparatide Comparator Trial In Vertebral Endpoints; ALN, alendronate; PBO, placebo; SC, subcutaneously; TPTD, teriparatide. From Bone HG, Cosman F, Miller PD, et al. ACTIVExtend: 24 months of alendronate after 18 months of abaloparatide or placebo for postmenopausal osteoporosis. *J Clin Endocrinol Metab.* 2018;103(8):2949–2957 (11) under the CC-BY license.

The designs of ACTIVE and ACTIVExtend have been previously described (10, 11, 14). Briefly, participants in ACTIVE were randomly assigned to receive 1:1:1 double-blind abaloparatide 80 μg or matching placebo given subcutaneously daily, or open-label teriparatide 20 μg subcutaneously daily for 18 months. During an off-treatment period of approximately 1 month (up to 40 days) following the completion of ACTIVE, eligible participants from the abaloparatide or placebo arms were recruited and provided consent for inclusion in ACTIVExtend. Participants enrolled in ACTIVExtend received alendronate 70 mg weekly for 24 months (Fig. 1). The primary endpoint both of ACTIVE and ACTIVExtend was the proportion of participants who experienced 1 or more incidents of new morphometric vertebral fracture.

ACTIVE and ACTIVExtend both were conducted according to the ethical principles contained in the Declaration of Helsinki and the recommendations of Good Clinical Practice guidelines as well as all applicable local regulatory and ethical requirements. All participants provided written informed consent to participate in the studies.

### Study analyses

New morphometric vertebral fractures were determined by spinal radiographs taken at baseline and at the end of treatment in ACTIVE and ACTIVExtend, as previously described (10). Radiographs were assessed by blinded, independent radiologists (Bioclinica-Synarc). Nonvertebral and clinical fractures, which comprise the majority of fragility fractures (15, 16), were secondary and exploratory endpoints of ACTIVE, respectively, and exploratory endpoints in ACTIVExtend. Nonvertebral fractures (initially self-reported and verified from source documents) were fractures that excluded those of the spine, sternum, patella, toes, fingers, skull, and face, and those associated with high trauma. High trauma was defined as a fall from a height equal to or higher than a stool, chair, or the first rung of a ladder. Clinical fractures were all fractures requiring a patient to seek medical care (including clinical spine), regardless of the level of trauma.

### Outcome measures

To evaluate the relative antifracture efficacy of abaloparatide vs alendronate as initial treatment, we compared the vertebral fracture incidence in participants enrolled in ACTIVExtend who received abaloparatide based on baseline and endpoint radiographs after 18 months of treatment during ACTIVE (abaloparatide group) and 24 months of alendronate treatment during ACTIVExtend (placebo/alendronate group) (Fig. 1). To account for differences in treatment duration, the fracture rate incidence was calculated using the number of events per 100 patient-years. Comparison between treatment groups was based on the Poisson model adjusted for age category (<65, 65-<75, ≥75 years). The incidence of vertebral fracture was calculated during the treatment periods in the modified intent-to-treat (mITT) populations from ACTIVExtend, which included all participants both with pretreatment and postbaseline spine x-rays. The incidences of nonvertebral and clinical fractures were calculated in the ITT populations from ACTIVExtend during 18 months of treatment with each therapy.

Patient characteristics reported at baseline of ACTIVE (abaloparatide group) and ACTIVExtend (placebo/alendronate group) were reported with corresponding *P* values calculated using a t test or chi-square test. Mean adherence to treatment was calculated accordingly for each treatment arm: (number of doses delivered/overall exposure duration) × 100.

## Results

### Participant characteristics

Participant characteristics for ACTIVE and ACTIVExtend have been previously reported (10, 11). Briefly, a total of 2463 participants were enrolled in ACTIVE, including 821 in the placebo group and 824 in the abaloparatide group. Participant characteristics were balanced at ACTIVE baseline. A total of 1243 participants (637 in the placebo group and 606 in the abaloparatide group) completed the ACTIVE study, and 581 from the placebo and 558 from the abaloparatide group were subsequently treated with alendronate in ACTIVExtend (11). Participants from the former abaloparatide and placebo groups who entered ACTIVExtend were well matched for baseline characteristics and were generally representative of the overall ACTIVE population (Table 1) (11). High level of treatment adherence was observed in both groups in ACTIVE and ACTIVExtend (ACTIVE: mean: 98.1% abaloparatide, 98.7% placebo; ACTIVExtend: 98.6% abaloparatide/alendronate, 97.6% placebo/alendronate).

**Table 1.** Comparison of Baseline Characteristics Between Alendronate- and Abaloparatide-Treated Participants

Baseline Characteristic	Abaloparatide Group at ACTIVE Study Baseline N = 558	Placebo/Alendronate Group at ACTIVExtend Study Baseline N=581	*P*
Age, y			
Mean y (SD)	68.6 (6.5)	70.1 (6.3)	<.001
<65, n (%)	106 (19.0)	86 (14.8)	.032
65 to <75, n (%)	351 (62.9)	359 (61.8)	
≥75, n (%)	101 (18.1)	136 (23.4)	
Race, n (%)			
White	433 (77.6)	447 (76.9)	.665
Asian	101 (18.1)	106 (18.2)	
Black or African American	19 (3.4)	18 (3.1)	
Other	5 (0.9)	10 (1.7)	
Ethnicity, n (%)			
Hispanic or Latino	124 (22.2)	139 (23.9)	.496
Not Hispanic or Latino	434 (77.8)	442 (76.1)	
Mean y since menopause (SD)	20.4 (8.2)	21.3 (7.9)	.042
Geographic region, n (%)			
North America	9 (1.6)	7 (1.2)	.915
South America	145 (26.0)	157 (27.0)	
Europe	305 (54.7)	312 (53.7)	
Asia	99 (17.7)	105 (18.1)	
Mean body mass index, kg/m^2^ (SD)	24.9 (3.5)	25.0 (3.6)	.672
Prevalent vertebral fracture at baseline, n (%)	123 (22.0)	140 (24.1)	.402
At least one prior nonvertebral fracture, n (%)	272 (48.7)	293 (50.4)	.570
Mean BMD T-score (SD)			
Lumbar spine	–2.88 (0.86)	–2.87 (0.87)	.947
Total hip	–1.88 (0.72)	–1.93 (0.76)	.312
Femoral neck	–2.14 (0.62)	–2.20 (0.70)	.187

Some data in this table were originally reported in Bone HG, Cosman F, Miller PD, et al. ACTIVExtend: 24 months of alendronate after 18 months of abaloparatide or placebo for postmenopausal osteoporosis. *J Clin Endocrinol Metab*. 2018;103(8):2949–2957 (11) under the CC-BY license.

Abbreviations: ACTIVE, Abaloparatide Comparator Trial In Vertebral Endpoints; BMD, bone mineral density.

### New vertebral fractures

**Figure 2.**
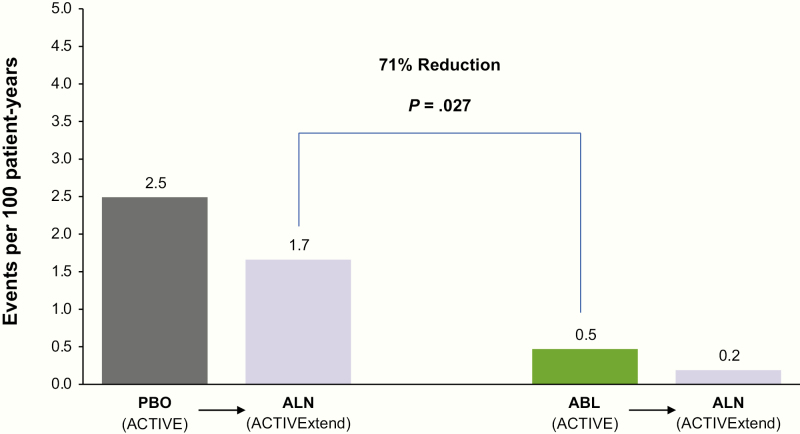
Comparison of new vertebral fracture event rate between alendronate- and abaloparatide-treated participants (ACTIVExtend mITT population), showing the reduction of new vertebral fractures in the placebo/alendronate (gray bar) and abaloparatide (green bar) groups from ACTIVE and in participants from each of these groups treated with alendronate (purple bars) during ACTIVExtend. ABL, abaloparatide; ACTIVE, Abaloparatide Comparator Trial In Vertebral Endpoints; ALN, alendronate; mITT, modified intent-to-treat; PBO, placebo.

The rate of new vertebral fractures during ACTIVE and ACTIVExtend is shown in Table 2 and Fig. 2. Women treated with abaloparatide in ACTIVE experienced fewer vertebral fractures compared to women treated with alendronate in ACTIVExtend. Specifically, the fracture rate during the abaloparatide treatment phase in ACTIVE treatment was 0.47 per 100 patient-years, whereas the fracture rate during the alendronate treatment phase in ACTIVExtend was 1.66 per 100 patient-years (relative risk reduction [RRR] 71%, *P* = .027). Of note, the vertebral fracture rate in the alendronate-treated women represented a reduction compared to the rate during the placebo-treatment phase (2.49 fractures per 100 patient-years), though the comparison did not meet statistical significance (RRR 32%; *P* = .22). A sensitivity analysis that included all participants who enrolled in ACTIVE, including those who did not enroll in ACTIVExtend, showed a vertebral fracture rate of 3.15 per 100 patient-years with placebo treatment during ACTIVE, and 1.66 per 100 patient-years during alendronate treatment in the placebo/alendronate arm in ACTIVExtend, corresponding to a 1.48 per 100 patient-years reduction after transition from placebo to alendronate (RRR 47% *P* = .031).The vertebral fracture rate in the abaloparatide-treated women was also lower after the transition to alendronate (0.19 per 100 patient-years) and again did not meet statistical significance (RRR 64%; *P* = .22).

**Table 2.** Comparison of Fracture Rates Between Alendronate- and Abaloparatide-Treated Participants

Nonvertebral and Clinical Fracture Event Rate per 100 Pt-Years Over Study Treatment (ACTIVExtend ITT Population)^a^					
	Abaloparatide N = 558	Placebo/Alendronate N = 581	Risk Difference (95% CI)	Risk Ratio (95% CI)	*P*
Total follow-up, pt-years	866.1	847.2			
Nonvertebral fracture event rate per 100 pt-years (No. of events)	1.39 (12)	2.36 (20)	1.08 (–2.40 to 0.24)	0.56 (0.27 to 1.14)	.108
No. of patients with nonvertebral fractures	12	19			
Clinical fracture event rate per 100 pt- years (No. of events)	2.54 (22)	2.83 (24)	–0.50 (–2.04 to 1.05)	0.83 (0.46 to 1.48)	.531
No. of patients with clinical fractures	16	21			
New Vertebral Fracture Event Rate per 100 Pt-Years Over Study Treatment (ACTIVExtend mITT Population)^b^					
	Abaloparatide N = 544	Placebo/Alendronate N = 568	Risk Difference (95% CI)	Risk Ratio (95% CI)	*P*
Total follow-up between baseline and last x-ray dates, pt-years	850.9	1085.4			
New vertebral fracture event rate per 100 pt-years (No. of events)	0.47 (4)	1.66 (18)	–1.14 (–2.02 to –0.26)	0.29 (0.10 to 0.87)	.027
No. of patients with new vertebral fractures	3	16			

Treatment comparison is based on the Poisson regression model adjusted by age category (<65, 65-<75, ≥75 years). Age-adjusted rate difference was calculated using the Mantel-Haenszel method.

Abbreviations: ACTIVE, Abaloparatide Comparator Trial In Vertebral Endpoints; CI, confidence interval; ITT, intent-to-treat; mITT, modified intent-to-treat; Pt-years, patient-years.

^a^Study drug treatment duration for abaloparatide is 18 months in ACTIVE. Nonvertebral and clinical fracture follow-up time is cut at 18 months.

^b^Placebo/Alendronate treatment duration is 24 months in ACTIVExtend.

Nonvertebral and clinical fracture rates during the first 18 months of treatment with abaloparatide and alendronate are shown in Table 2. The comparisons were based on a limited number of fractures and were not statistically significant.

## Discussion

To date, no studies have prospectively compared the impact of initial treatment with abaloparatide to that of a bisphosphonate on fracture incidence in patients with osteoporosis. In this post hoc analysis, we indirectly compared the antifracture efficacy of abaloparatide and alendronate treatment in the previously described ACTIVExtend cohort (11, 14) because these groups were well matched, and all patients were eligible for alendronate treatment in ACTIVExtend. Although the group initially treated with placebo began alendronate therapy approximately 18 months later than the initial random assignment to abaloparatide or placebo and a corresponding age difference was observed between ACTIVE and ACTIVExtend baseline characteristics, between-treatment comparisons were adjusted for age category. No interaction was observed between age and treatment.

Comparison of the fracture rates from the abaloparatide group from ACTIVE with the group that received alendronate in ACTIVExtend following placebo treatment in ACTIVE suggests that, for postmenopausal women with osteoporosis, abaloparatide may provide greater risk reduction for vertebral fracture incidence than alendronate.

A similar reduction in vertebral fractures has been observed in previous studies examining the impact of alendronate treatment on postmenopausal women with osteoporosis as was seen in our analysis (17, 18). Specifically, in our analysis, the vertebral fracture rate declined by 32% to 47% in participants treated with alendronate, whereas risk of vertebral fracture was 47% lower in alendronate-treated participants in the Fracture Intervention Trial 1 (FIT1) study (17) and 44% lower in alendronate-treated participants in FIT2 (18). The incidence for nonvertebral fractures declined by approximately 18% in participants initially treated with placebo and then switched to alendronate in our study, compared with a 20% reduction in risk of nonvertebral fractures in FIT1 (17) and a 12% reduction in FIT2 (18), although results were not statistically significant in any of the 3 studies.

Several previous studies have compared surrogate markers of fracture risk, such as BMD and markers of bone turnover, to assess differences between antiresorptive vs anabolic agents (19–21). In addition, 2 clinical trials that examined the risk of fracture in participants treated with the anabolic drug teriparatide or oral bisphosphonates as a secondary or exploratory outcome showed reduced risk of new vertebral fractures with teriparatide (22, 23). Recently, the first head-to-head comparison of the effects of an anabolic agent (teriparatide) and an antiresorptive drug (risedronate) on fracture incidence as a primary outcome was conducted (12). This study (VERO), by Kendler et al, showed statistically significantly lower risk of new vertebral and clinical fractures in postmenopausal women treated for 24 months with teriparatide compared with risedronate. Our findings add to this body of evidence and suggest other anabolic agents may also have a beneficial effect on fracture incidence compared with bisphosphonates, and support the need for additional studies directly comparing abaloparatide treatment with alendronate and other antiresorptive treatments. In contrast to the VERO trial, individuals enrolled in ACTIVE had not received significant antiresorptive therapy within 5 years of the study, so the present results are more reflective of response to initial therapy.

Limitations of this post hoc analysis include relatively small numbers of fractures in the abaloparatide and alendronate groups, as well as any potential effect of the 18-month time offset in the comparison between the abaloparatide population from ACTIVE vs the alendronate population from ACTIVExtend.

The results of this post hoc analysis suggest that initial treatment of postmenopausal women with osteoporosis with abaloparatide may provide benefits in terms of fracture risk reduction vs alendronate alone. Additional studies are warranted to confirm these findings.

## Abbreviations

ABL: abaloparatide
ACTIVE: Abaloparatide Comparator Trial In Vertebral Endpoints
ALN: alendronate
BMD: bone mineral density
CI: confidence interval
ITT: intent-to-treat
mITT: modified intent-to-treat
PBO: placebo
Pt-years: patient-years
RRR: relative risk reduction
SC: subcutaneous
TPTD: teriparatide

## Additional Information

***Disclosure Summary:*** BZL has received research funding from Amgen and Lilly and has participated on scientific advisory boards for Amgen, Lilly, and Radius Health, Inc. RSB has participated on a scientific advisory board for Radius Health, Inc. GH was an employee at Radius Health, Inc at the time of manuscript development but is no longer employed there. He is a scientific advisory board member and consultant for Radius Health, Inc. BM is an employee of and owns company stock in Radius Health, Inc. MH was an employee of Radius Health, Inc at the time of manuscript development but is no longer employed there.

***Data Availability:*** Restrictions apply to the availability of data generated or analyzed during this study to preserve patient confidentiality or because they were used under license. The corresponding author will on request detail the restrictions and any conditions under which access to some data may be provided.
