# ERRATUM for “Effect of Abaloparatide vs Alendronate on Fracture Risk Reduction in Postmenopausal Women With Osteoporosis”

**DOI:** 10.1210/clinem/dgaa301

**Published:** 2020-06-09

In the above-named article by Leder BZ, Mitlak B, Hu M, Hattersley G, Bockman RS (*J Clin Endocrinol Metab*. 2020;105(3):938–943; doi: 10.1210/clinem/dgz162), the following error occurred during the production process in the published paper:

In Figure 2, the green bar label (0.5) was incorrectly shown as “PBO”. The correct label for this bar is “ABL” (abaloparatide). The figure has been replaced in the article.

